# Immunosuppressants in women with repeated implantation failure in assisted reproductive techniques: a systematic review and meta-analysis

**DOI:** 10.61622/rbgo/2024rbgo70

**Published:** 2024-09-18

**Authors:** Ana Clara Felix de Farias Santos, Fernanda Valeriano Zamora, Lubna Al-Sharif, Kush Sehgal, Deyvid Vieira Silva Cavalcante, Sarah Hasimyan Ferreira, Pedro Henrique Costa Matos da Silva

**Affiliations:** 1 Universidade Cidade de São Paulo São Paulo SP Brazil Universidade Cidade de São Paulo, São Paulo, SP, Brazil.; 2 Universidade Federal de Minas Gerais Belo Horizonte MG Brazil Universidade Federal de Minas Gerais, Belo Horizonte, MG, Brazil.; 3 An-Najah National University Nablus WB Palestine An-Najah National University, Nablus, WB, Palestine.; 4 Teerthanker Mahaveer University Moradabad UP India Teerthanker Mahaveer University, Moradabad, UP, India.; 5 Universidade CEUMA São Luis MA Brazil Universidade CEUMA, São Luis, MA, Brazil.; 6 Departamento de Ginecologia e Obstetrícia Faculdade de Medicina Universidade Federal de Goiás Goiânia GO Brazil Departamento de Ginecologia e Obstetrícia, Faculdade de Medicina, Universidade Federal de Goiás, Goiânia, GO, Brazil.

**Keywords:** Repeated implantation failure, Reproductive techniques, assisted, Cyclosporine A, Prednisone, Prednisolone Immunosupressive agents, Reproduction

## Abstract

**Objective:**

To compare outcomes in patients with repeated implantation failure undergoing Intracytoplasmic Sperm Injection/In vitro fertilization (IVF/ICSI) plus immunosuppressants such as prednisolone, prednisone, or cyclosporine A versus the use of IVF/ICSI alone.

**Data source:**

Databases were systematically searched in PubMed, Cochrane, and Embase databases in September 2023.

**Study Selection:**

Randomized clinical trials and observational studies with the outcomes of interest were included.

**Data collect:**

We computed odds ratios (ORs) for binary endpoints, with 95% confidence intervals (CIs). Heterogeneity was assessed using I^2^ statistics. Data were analyzed using Review Manager 5.4.The main outcomes were live birth, miscarriage, implantation rate, clinical pregnancy, and biochemical pregnancy.

**Data synthesis:**

Seven studies with 2,829 patients were included. Immunosuppressive treatments were used in 1,312 (46.37%). Cyclosporine A improved implantation rate (OR 1.48; 95% CI 1.01-2.18) and clinical pregnancy (1.89, 95% CI 1.14-3.14). Compared to non-immunosuppressive treatment, prednisolone and prednisone did not improve live birth (OR 1.13, 95% CI 0.88-1.46) and miscarriage (OR 1.49, 95% CI 1.07-2.09). Prednisolone showed no significant effect in patients undergoing IVF/ICSI, clinical pregnancy (OR 1.34; 95% CI 0.76-2.36), or implantation rate (OR 1.36; 95% CI 0.76-2.42).

**Conclusion:**

Cyclosporine A may promote implantation and clinical pregnancy rates. However, given the limited sample size, it is important to approach these findings with caution. Our results indicate that prednisolone and prednisone do not have any beneficial effects on clinical outcomes of IVF/ICSI patients with repeated implantation failure.

**PROSPERO:**

CRD42023449655

## Introduction

Recurrent implantation failure (RIF) is defined as the condition in which women undergo three unsuccessful attempts of in vitro fertilization (IVF), despite utilizing embryos of good quality. Various factors contribute to the likelihood of recurrent implantation failure, including advancing maternal age, dual smoking habits of both parents, having a higher body mass index, and experiencing elevated stress levels.^(1-5)^

Previous studies have indicated that the inability of apparently viable embryos to implant may be attributed to abnormalities in cellular adhesion molecules, imbalances in the network of cytokines, or excessive activity of uterine natural killer cells.^(2-8)^

There is a persistent interest in utilizing immune-suppressant corticosteroid medications such as prednisolone for treating infertility in women experiencing repeated IVF failure and recurrent miscarriage. This proposition is grounded in the hypothesis that the maternal immune system might mount a response against the embryo’s tissue.^(9-14)^Prednisolone and prednisone are corticosteroids employed to mitigate inflammation and alleviate an overactive immune system.^(15,16)^Cyclosporine A (CsA), a calcineurin inhibitor, is another immunosuppressive medication commonly used to prevent organ rejection in transplant recipients and treat certain autoimmune conditions. Additionally, Cyclosporine is employed in the treatment of severe psoriasis and severe rheumatoid arthritis.^(17)^Recently, both immunosuppressive medications have increasingly been used in the treatment of RIF to enhance clinical outcomes in IVF/ICSI.^(14)^

Nevertheless, the effectiveness of prednisolone, prednisone, and CsA for women with a history of RIF remains controversial. Previous studies reported positive outcomes associated with immunosuppressant use in IVF/ICSI cycles,^(7,8)^while others suggest minimal or no impact on pregnancy outcomes.^(9-14)^The primary objective of this investigation is to assess the potential benefits of employing immunosuppressant drugs in patients with a history of RIF undergoing in vitro fertilization, in comparison to the standard of care, to determine whether the utilization of these immunosuppressants provides any practical advantages.

## Methods

This systematic review and meta-analysis followed the recommendations of the Preferred Reporting Items for Systematic Reviews and Meta-Analysis (PRISMA) statement.^(4)^

### Inclusion criteria and data extraction

Studies meeting the following inclusion criteria were included: (1) randomized clinical trials or nonrandomized cohort studies; (2) inclusion of women with RIF; (3) presence of at least one arm incorporating immunosuppressants; and (4) inclusion of a control group (either placebo-controlled or standard of care-controlled). There were no restrictions on the date of publication or language. Exclusion criteria encompassed: (1) absence of intervention or control group; (2) overlapping populations; (3) lack of at least one primary outcome of interest (live birth, miscarriage, implantation rate, clinical pregnancy, biochemical pregnancy).

Three authors independently reviewed the reports to determine their eligibility through consensus. All potentially relevant articles were reviewed by reading the full texts to identify eligible trial reports after excluding irrelevant studies. Data were manually extracted from eligible full-text articles.

### Data sources and search strategy

MEDLINE (via PubMed), Embase, and the Cochrane Central Register of Controlled Trials databases were systematically searched in September 2023. Additionally, references of eligible papers and systematic reviews were also searched for additional studies. A comprehensive search strategy was employed to ensure the identification of all relevant trials. The search strategy included combinations of Medical Subject Heading (MeSH) terms, advanced search terms, and Boolean operators: “Prednisone OR prednisolone OR methylprednisolone OR dexamethasone hydrocortisone OR cortisone OR corticosteroid OR glucocorticoid OR cyclosporine AND (Implantation Failure) AND Recurrent OR Repeated”.

### Study selection and subgroup analysis

Three investigators independently collected data from the appropriate studies and recorded it on specialized spreadsheets. The following information was extracted from eligible studies: (1) study characteristics - authors, study design, sample size per group, study population; (2) patient characteristics - mean age, country, body mass index, comorbidities; (3) outcomes - live birth, miscarriage, implantation rate, clinical pregnancy, and biochemical pregnancy; and (4) subgroups - prednisolone and CsA.

To assess whether an individual study exerted a stronger influence on the pooled results, a leave-one-out sensitivity analysis was conducted for live birth, miscarriage, implantation rate, clinical pregnancy, and biochemical pregnancy.

### Risk of bias assessment

Two authors independently evaluated the risk of bias, and disagreements were resolved through consensus after discussing reasons for discrepancies with the first author (A.C.F.F.S). The risk of bias was assessed using the Cochrane tool for assessing risk of bias 1 (Rob1) for non-randomized studies and risk of bias 2 (Rob2) for randomized studies, following the recommendations from the Cochrane Handbook for Systematic Reviews of Interventions.^(5)^Accordingly, a “high risk” of bias designation was assigned to studies demonstrating a high risk of bias on any domain of the Robins I or Rob2 tool. Studies presenting some concerns on any domain were labeled as having “some concerns,” while studies with a low risk of bias were identified as such.^(3)^

Publication bias was evaluated through visual inspection of funnel plots. Since the number of studies included in this meta-analysis was less than ten, no attempt was made to quantitatively assess small studies or publication bias.^(6)^

### Statistical analysis

The data were analyzed using the Cochrane Review Manager software (RevMan 5.4). Binary endpoints were summarized utilizing the Mantel-Haenszel test with a random-effects model, anticipating low heterogeneity for the measured outcomes. The odds ratio (OR) and 95% confidence interval (CI) were employed as measures of the effect size. Heterogeneity was evaluated using Cochrane’s Q statistic and Higgins and Thompson’s I^2^ statistics. The significance of the pooled ratios was determined by the Z test, and a p-value below 0.05 was considered statistically significant.

## Results

### Study selection and characteristics

A total of 4,335 studies were screened, and after removing duplicates, 122 studies underwent full-text review. Ultimately, 7 articles met the inclusion criteria. The included studies comprised 2 randomized trials, 1 quasi-randomized trial, 3 retrospective cohort studies, and 1 cohort involving 2,829 patients, with 1,312 (46.37%) in the intervention group and 1,517 (53.62%) in the control group. The follow-up duration ranged from 7 years to 11 months. Figure 1 illustrates the overall protocol for the study and provides details on the number of studies excluded. The mean age of participants ranged from 30.8 to 35.1 years, and the mean BMI ranged from 21.4 to 27.3. Four of the included studies^(8,10,12,13)^evaluated the use of prednisolone in the intervention group. Only two retrospective cohorts^(7,11)^studied the effects of CsA, and one study^9^ evaluated prednisone. Only one study had vaginal progesterone as a control group.^(12)^Chart 1 summarizes the main characteristics of the included studies.

**Figure 1:**
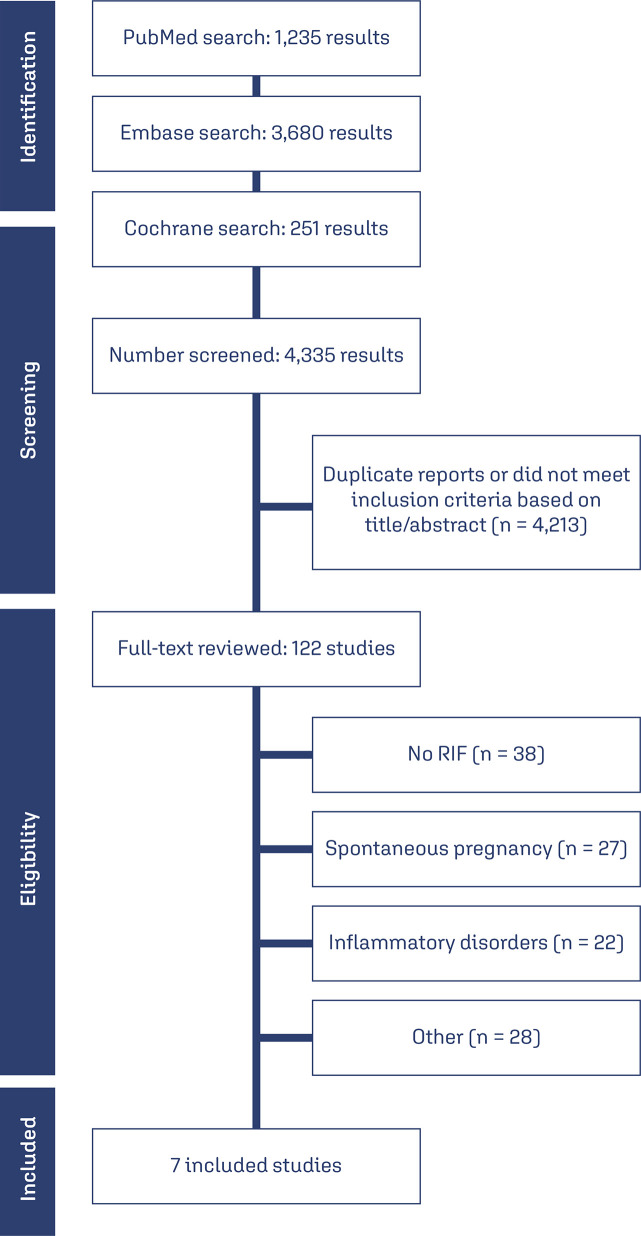
Study screening and selection

**Chart 1:** Baseline characteristics of included studies

Study, (year) reference	Study design	Country	Nº of patients IS/NIS	Age (years)	Immunosuppressant	BMI IS/NIS	Tubal factor etiology	Pregnancy outcome	Inclusion criteria
Cheng et al. (2022)^(7)^	RCS	China	58/ 66	IS: 32.62 ± 0.46 NIS: 32.80 ± 0.45	CsA 50mg	IS: 22.3 ± 0.4 NIS: 21.4 ± 0.3	IS: 3 (5.1) NIS: 4 (6.0)	Live birth Miscarriage Implantation rate Clinical pregnancy	URIF patients
Fawzy and El-Refaeey (2014)^(8)^	Quasi-RCT	Egypt	145/ 142	IS: 30.8 ± 4.9 NIS: 31.3 ± 5.8	Prednisolone 20mg + LMWH	IS: 27.3 ± 4.5 NIS: 26.1 ± 5.1	NA	Implantation rate Clinical pregnancy	Patients with previously unexplained, failed one or two ICSI attempts
Sun et al.(2023)^(9)^	RCT	China	357/ 358	IS: 31.1 ± 3.6 NIS: 31.5 ± 3.5	Prednisone 10mg	IS: 22.7 (3.1) NIS: 22.7 (3.1)	IS: 156 (43.7) NIS: 141 (39.4)	Live birth Clinical pregnancy Biochemical pregnancy Implantation rate Miscarriage	Women who undergone ≥ 2 ETC but did not achieve a clinical pregnancy
Ubaldi et al. (2002)^(10)^	RCT	Italy	159/ 156	IS: 33.1 ± 3.7 NIS: 32.7 ± 3.4	Prednisolone 10mg	NA	NA	Clinical pregnancy Implantation rate	Patients with ≤ 3 ICSI attempts
Qu et al.^**^ (2021)^(11)^	RCS	China	58/ 120	IS: 32.0 NIS: 31.5	CsA 50mg	IS: 22.5 ± 3.3 NIS: 22.8 ± 3.5	IS: 63.2 (24) NIS: 60.2 (50)	Implantation rate Clinical pregnancy Biochemical pregnancy Take-home baby rate	Patients with unexplained transfer failure in FET cycles
Aslan et al^*^ (2023)^(12)^	RCS	Turkey	478/617	IS: 32.2 ± 4.4 NIS: 32.1 ± 4.3	LMWH+AAS+ Prednisolone 16mg	IS: 26.2 ± 5.3 NIS: 25.5± 4.8	IS: 28 (6.0) NIS: 43 (7.0)	Live birth Miscarriage	Patients with at least 2 previous IF
Siristatidis et al. (2018)^(13)^	RCS	Egypt	57/ 58	IS: 35.1 ± 0.7 NIS: 34.1 ± 0.7	Prednisolone 5mg + LMWH	IS: 25.0 ± 0.5 NIS: 24.5 ± 0.4	IS: 7 (12.3) NIS: 8 (13.8)	Clinical pregnancy Biochemical pregnancy Miscarriage Live birth	RIF patients

Values are mean, SD or median (interquartile range); AAS - aspirin BMI: body mass index; BP - Biochemical pregnancy; CL - Clinical Pregnancy; CsA - cyclosporine A; ETC - embryo transfer cycles; FET - frozen-thawed embryo transfer; IS - intervention group with immunosuppressant; ICSI - intracytoplasmic sperm injection; IF - implantation failure; LMWH - low dose molecular heparin; MG - milligram; NA - not available; NIS - control group without immunosuppressant; Quasi-RCT - quasi-randomized trial; RCS - retrospective cohort study; RCT - randomized controlled trial; RIF - repeated implantation failures; URIF - unexplained recurrent implantation failure. ^*^Age at oocyte pick-up; ^**^Age at oocyte retrieval

### Pooled analysis of all studies

In comparison to basic treatment, CsA improved Implantation rate and clinical pregnancy (Figure 2) in patients with RIF, while no significant differences were observed in live birth, miscarriage, and biochemical pregnancy between the treatments (Figure 3). Prednisolone and prednisone had no benefit on implantation rate and clinical pregnancy among patients undergoing IVF/ICSI (Figure 2). Live birth and miscarriage also did not have a significant difference between the groups (Figures 3A and 3B). Furthermore, prednisone and prednisolone did not reduce the rate of biochemical pregnancy (OR 1.40; 95% CI 0.83–2.37) when compared to basic treatment (Figure 3C).

**Figure 2:**
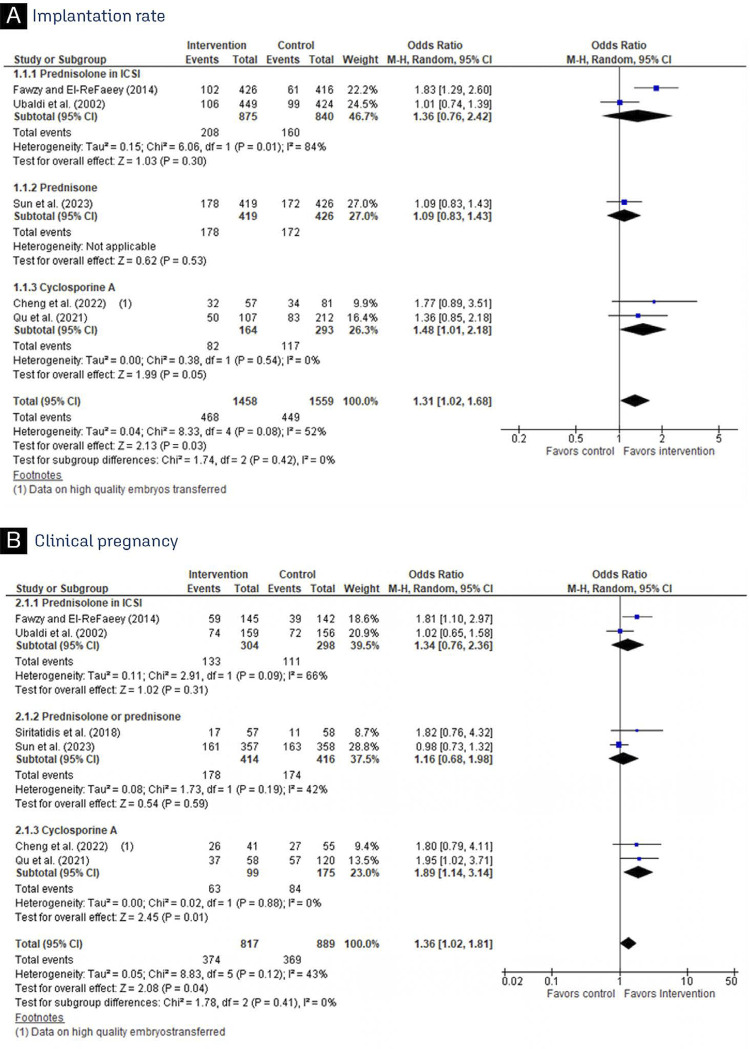
Forest plot of studies examining outcomes between patients receiving immunosuppressant or standard treatment: (A) Implantation rate; (B) Clinical pregnancy

**Figure 3:**
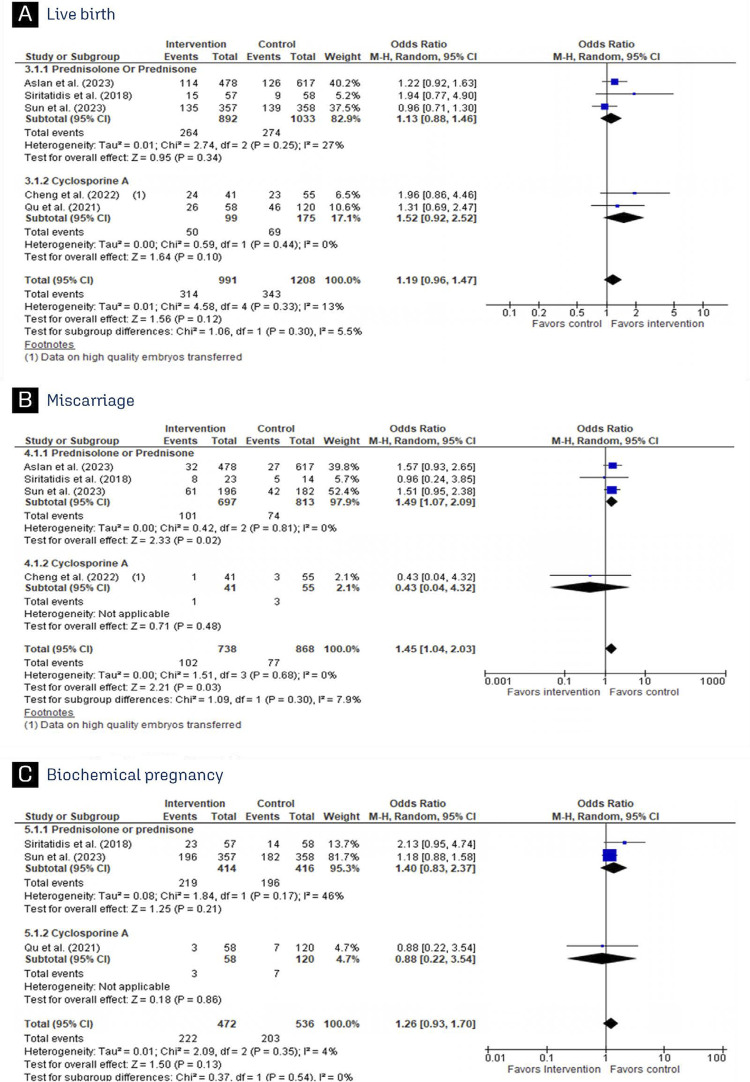
Forest plot of studies examining outcomes between patients receiving immunosuppressant or standard treatment: (A) Live birth; (B) Miscarriage; (C) Biochemical pregnancy

### Sensitivity analysis

Leave-one-out sensitivity analyses were performed for all endpoints, revealing no substantial alterations in the statistical significance of pooled effect sizes. Changes in heterogeneity were noted when removing Siristatidis et al.^(13)^ and Sun et al.^(9)^ trials in the outcome of live birth, with a 23-0% reduction in both studies. The decrease in heterogeneity was likely attributed to methodological factors, including study design and population characteristics. Heterogeneity remained stable when omitting Aslan et al.,^(12)^ Siristatidis et al.^(13)^ and Sun et al.,^(9)^ which examined the effects of Prednisolone and Prednisone on miscarriage outcomes. The sensitivity analyses for the primary outcomes are presented in the Supplementary material Table 1S-5S.

### Risk of bias within studies and publication bias

**Figure 4:**
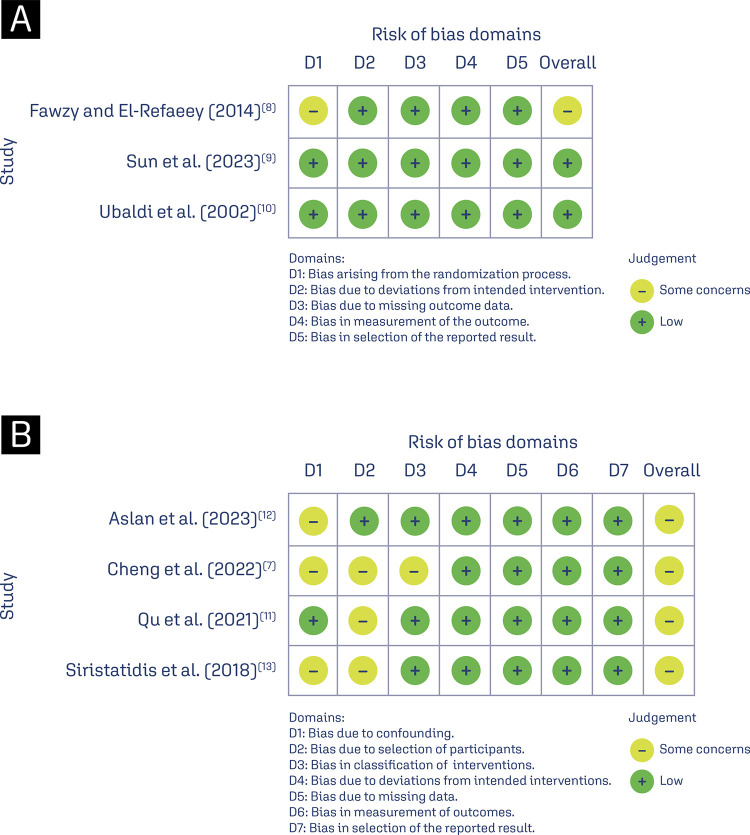
Risk of bias: (A) Critical appraisal of randomized controlled trials according to the Cochrane Collaboration tool for assessing risk of bias in randomized trials (Rob 2); (B) Traffic light diagram representing the critical appraisal of non- RCTs according to the Cochrane Collaboration’s tool for assessing the risk of bias in non- randomized trials (Robins-I)

The risk of individual within-study bias is represented in the Rob 2 and Robins-I traffic-light diagram (Figure 4). Fawzy and El-Refaeey^(8)^ were considered at risk for the randomization process, due to participants being allocated by alternation. The remaining RCTs were assigned a low risk of bias.^(9,10)^All cohorts raised some concerns regarding bias in at least one Robins-I assessment tool domain.^(7,11-13)^The analysis of funnel plots for the live birth and implantation rate outcomes suggests no evidence of serious publication bias. The funnel plots for the endpoints are available in Supplementary material Figure 1S, 2S.

## Discussion

This systematic review and meta-analysis, including two RCTs and five NRSI (4 RCS and 1 quasi-RCT), involving a total of 2,829 patients, aimed to evaluate the efficacy of immunosuppressants in women with recurrent implantation failure (RIF) undergoing in IVF/ICSI. The findings of this study revealed the following associations: 1) CsA improved the implantation rate (OR 1.48; 95% CI 1.01–2.18) and clinical pregnancy (1.89, 95% CI 1.14–3.14) when compared with basic treatment; 2) Prednisone did not show improvement in live birth, miscarriage, and biochemical pregnancy. 3) Prednisolone showed no significant effect in patients undergoing IVF/ICSI, and there was no difference in live birth and miscarriage.

The analysis demonstrated a significant impact of total immunosuppression on pregnancy outcomes when compared to placebo or control groups in RIF patients. Particularly, there is noteworthy variability in the treatment outcomes when comparing the use of glucocorticoids (prednisone or prednisolone) versus CsA. The analysis highlights the potential of CsA in enhancing both embryo implantation and clinical pregnancy rates, without any apparent detrimental effects when compared to glucocorticoids. Conversely, biochemical pregnancy success rate and neonatal live birth rate were observed to be negatively affected by immunosuppressants but not by placebo/control use.^(18-22)^

Regarding CsA, its immunosuppressant activity is due to its binding with cyclophilin, one of the main cytosolic proteins of NK cells, and forming a complex which hinders with the production of lymphokines including IL-2, IFN-γ, and TNF-α as well as lymphocyte proliferation.^(22,23)^Additionally, CsA has proved its efficacy in crossing placenta with variable range of concentration in fetus (37 - 64%) when compared with maternal levels. The safety and efficacy potentials of CsA were addressed in children treated for vernal keratoconjunctivitis, while some studies reported no congenital defects despite the increased risk of low birth weight and possible preterm birth.^(24-26)^ Only two studies^(7,11)^addressed the CsA effectiveness in pregnancy outcomes in RIF patients, especially after embryo transfer, without raising the chance of pediatric or obstetric complications.

In addition, CsA showed a significant improvement in rates of embryo implantation, clinical pregnancy, and neonatal live birth among RIF patients who failed to respond to prior therapies with prednisone, aspirin, LMWH, IVIG, and lymphocyte immunotherapy, and who also suffered from antiphospholipid syndrome. An initial cohort study which observed CsA AND progesterone as a control suggested CsA effectiveness in elevating successful pregnancy rate by 76.92% was due to autoantibodies levels reduction.^(27)^ Besides, a randomized control trial highlighted CsA potential towards lowering the CD57+ and CD56+ cells during luteal phase in women with RSA who also suffered from endometrial alloimmune dysfunction.^(28,29)^Currently, there is limited evidence available regarding the safety of CsA. However, it is generally deemed safe due to the positive outcomes observed in the subgroup treated with CsA during short-term therapy use, which lasted for a period of 12-14 days. This therapy began on the day of oocyte transfer and continued until the biochemical pregnancy testing stage.^(7,11)^

Notably, there is an increased rate of miscarriage in the overall estimate of glucocorticoids and CsA in our study. The lack of knowledge to the actual reasons behind RIF condition in our glucocorticoids-treated subgroup, when compared to control/placebo could be explained by the presence of excessive immune response of T helper 1 (Th1; including IFN-γ and TNF-α) at the time of implantation, and thus, the studied dosing regimens failed in supporting pregnancy outcomes, leading to a significant elevation of miscarriage rates and resulting in miscarried implantation, early loss of pregnancy, and also recurrent pregnancy loss.^(9,12,13,29,30)^Regarding the CsA-treated subgroup, there is a lack of studies addressing the CsA potential towards positive lowering effect of miscarriage rate, which may contribute to incomplete miscarriage rate assessment.

The duration of therapy as well as the timing of its initiation varied widely between studies, and this was represented in glucocorticoids regimen administration starting the day one of ovarian stimulation / pre-implantation, and extended differently as in Ubaldi et al. (2002)^(10)^ (4 weeks till pregnancy confirmation), and Sun et al. (2023)^(9)^ and Aslan et al. (2023)^(12)^ (12-16 weeks of gestation after pregnancy confirmation). However, Fawzy and El-Refaeey (2014)^(8)^ demonstrated two distinct times for prednisolone and adjuvant therapy at day one of ovarian stimulation and oocyte retrieval, respectively. The oocyte retrieval step was the point of CsA therapy initiation, which continued till the pregnancy was confirmed (12-14 days).^(7,11)^Also, the use of various adjuvant treatments, including glucocorticoid therapy, can have a significant impact on the immunosuppressive potential in patients with recurrent implantation failure (RIF) undergoing in vitro fertilization (IVF). However, the effectiveness of these treatments in improving the success rates of IVF procedures can vary. In two randomized studies, it was found that the administration of 80 mg of Aspirin may or may not lead to improvements in implantation and pregnancy rates for patients undergoing intracytoplasmic sperm injection (ICSI).^(20,31,32)^

Prior randomized and observational studies have supported the inefficacy of glucocorticoids, compared with placebo/ control, in improving the embryo implantation and/or pregnancy rates in routine IVF/ICSI recipients. Those findings were confirmed by using either high or low doses (methylprednisone; 60 mg for 4 days, and prednisolone; 10 mg/day, respectively), and as individual therapy / or in a combination with at least one of adjuvant therapies (aspirin/LMWH/hCG/antibiotics).^(15,18,19,21)^Also, neither the use of cryopreservation of embryos and frozen-thawed embryo transfer nor applying oocyte retrieval without further processing, succeeded in improving any of pregnancy outcomes in women with unexplained infertility before or with RIF in our current review.^(9,10,12,13)^

However, the possibility of selection bias, confounders, limited sample size of non-RCTs, and the use of anti-inflammatory dose of prednisone/prednisolone (10mg/day) in both RCTs and non-RCTs, might contribute to reducing the power of the results regarding outcomes improvement. On the contrary, and El-Refaeey (2014)^(8)^showed that higher doses of prednisolone (20 mg/day) could suppress the possible NK cell cytolytic activity and thus, consequent embryo-endometrial interaction can be minimized.

Our study has limitations. Firstly, we observed moderate to high heterogeneity in certain outcomes analyzed, such as implantation rate. However, we performed leave-one-out sensitivity analyses which yielded similar results after omitting each study from the analysis. Additionally, some studies included in our analysis exhibited bias in randomization or participant selection. Another limitation stems from the relatively small sample size of participants in the CsA studies. Furthermore, most of the included studies were retrospective cohorts, which increases the possibility of bias due to confounding factors.

## Conclusion

Although CsA has shown potential in enhancing implantation rate and clinical pregnancy, these findings are preliminary and further research on a larger scale is essential to validate these results and provide a more accurate assessment of its efficacy and implication. In this meta-analysis, the use of prednisone or prednisolone was not associated with improved live birth, implantation rate, and clinical pregnancy, as compared to the use of placebo or standard treatment. Furthermore, the use of glucocorticoids was associated with an increased risk of miscarriage. These findings question the effectiveness and safety of the glucocorticoids as a viable treatment option for individuals facing RIF.

## Figures and Tables

**Figure 1S:**
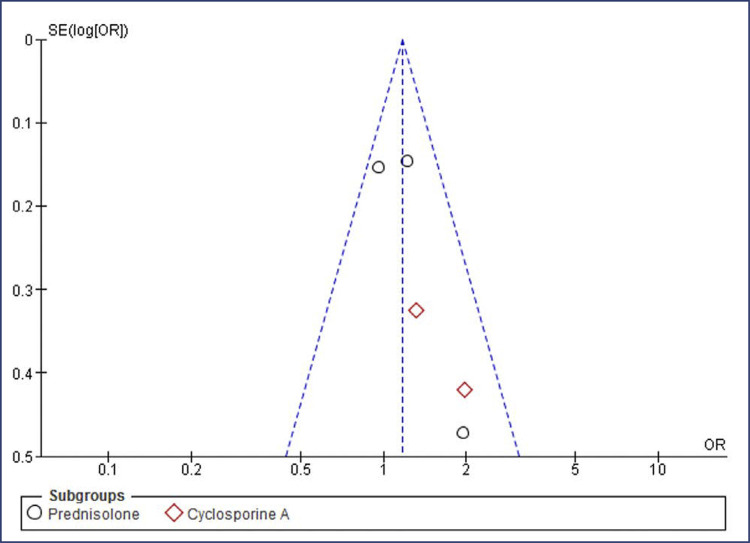
Funnel plots: Live birth

**Figure 2S:**
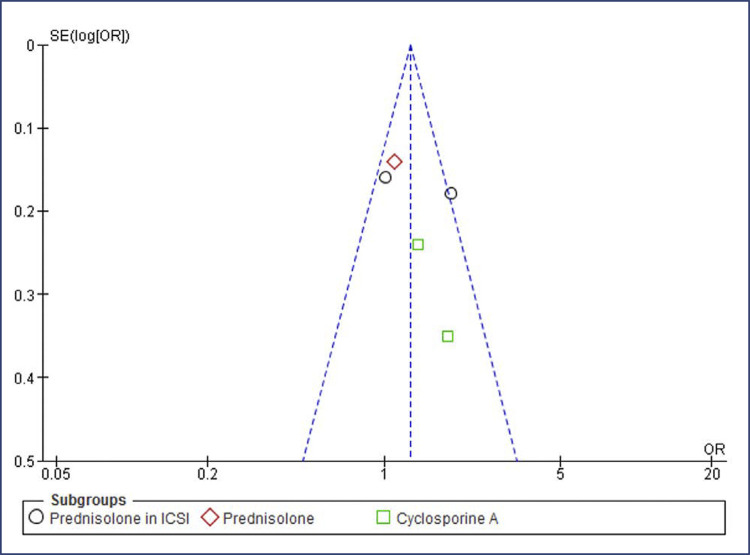
Funnel plots: Implantation rate

## Supplementary material

**Table 1S:** Leave-one-out sensitivity analysis for Live birth

Prednisolone			
Study	OR	95% CI	I^2^
Omitting Aslan et al. (2023)^12^	1.19	(0.63 - 2.24)	51%
Omitting Siristatidis et al. (2018)^(13)^	1.09	(0.86 - 1.38)	23%
Omitting Sun et al. (2023)^(9)^	1.27	(0.97 - 1.67)	0%
Including all studies	1.13	(0.88 - 1.46)	27%
Cyclosporine A			
Study	OR	95% CI	I^2^
Omitting Cheng et al. (2022)^(7)^	1.31	(0.69 - 2.47)	NA
Omitting Qu et al. (2021)^(11)^	1.96	(0.86 - 4.46)	NA
Including all studies	1.52	(0.92 - 2.52)	0%

NA - not applicable

**Table 2S:** Leave-one-out sensitivity analysis for Miscarriage

Prednisolone			
Study	OR	95% CI	I^2^
Omitting Aslan et al. (2023)^12^	1.44	(0.93 - 2.23)	0%
Omitting Siristatidis et al. (2018)^(13)^	1.53	(1.08 - 2.17)	0%
Omitting Sun et al. (2023)^(9)^	1.47	(0.90 - 2.41)	0%
Including all studies	1.49	(1.07 - 2.09)	0%

**Table 3S:** Leave-one-out sensitivity analysis for Implantation rate.

Prednisolone in ICSI			
Study	OR	95% CI	I^2^
Omitting Fawzy and El-Refaeey (2014)^(8)^	1.01	(0.74 - 1.39)	NA
Omitting Ubaldi et al. (2002)^(10)^	1.83	(1.29 - 2.60)	NA
Including all studies	1.36	(0.76 - 2.42)	84%
Cyclosporine A			
Study	OR	95% CI	I^2^
Omitting Cheng et al. (2022)^(7)^	1.36	(0.85 - 2.18)	NA
Omitting Qu et al. (2021)^(11)^	1.77	(0.89 - 3.51)	NA
Including all studies	1.48	(1.01 - 2.18)	0%

NA - not applicable

**Table 4S:** Leave-one-out sensitivity analysis for Clinical pregnancy

Prednisolone in ICSI			
Study	OR	95% CI	I^2^
Omitting Fawzy and El-Refaeey (2014)^(8)^	1.02	(0.65 - 1.58)	NA
Omitting Ubaldi et al. (2002)^(10)^	1.81	(1.10 - 2.97)	NA
Including all studies	1.34	(0.76 - 2.36)	66%
Prednisolone			
Study	OR	95% CI	I^2^
Omitting Siristatidis et al. (2018)^(13)^	0.98	(0.73 - 1.32)	NA
Omitting Sun et al. (2023)^(9)^	1.82	(0.76 - 4.32)	NA
Including all studies	1.16	(0.68 - 1.98)	42%
Cyclosporine A			
Study	OR	95% CI	I^2^
Omitting Cheng et al. (2022)^(7)^	1.95	(1.02 - 3.71)	NA
Omitting Qu et al. (2021)^(11)^	1.80	(0.79 - 4.11)	NA
Including all studies	1.89	(1.14 - 3.14)	0%

NA - not applicable

**Table 5S:** Leave-one-out sensitivity analysis for Biochemical pregnancy

Prednisolone in ICSI			
Study	OR	95% CI	I^2^
Omitting Siristatidis et al. (2018)^(13)^	1.18	(0.88 - 1.58)	NA
Omitting Sun et al. (2023)^(9)^	2.13	(0.95 - 4.74)	NA
Including all studies	1.40	(0.83 - 2.37)	46%

NA - not applicable
